# Comparative structural analysis on the mitochondrial DNAs from various strains of *Lentinula edodes*

**DOI:** 10.3389/fmicb.2022.1034387

**Published:** 2022-11-28

**Authors:** Sinil Kim, Hyerang Eom, Rutuja Nandre, Yeon Jae Choi, Hwayong Lee, Hojin Ryu, Hyeon-Su Ro

**Affiliations:** ^1^Department of BioMedical Bigdata (BK21) and Research Institute of Life Sciences, Gyeongsang National University, Jinju, South Korea; ^2^Department of Forest Science, Chungbuk National University, Cheong-ju, South Korea; ^3^Department of Biological Sciences and Biotechnology, Chungbuk National University, Cheong-ju, South Korea

**Keywords:** *Lentinula edodes*, mitochondrial DNA, evolution, intron, repeats

## Abstract

The evolution of mitochondria through variations in mitochondrial DNA (mtDNA) is one of the intriguing questions in eukaryotic cells. In order to assess the causes of the variations in mitochondria, the mtDNAs of the 21 strains of *Lentinula edodes* were assembled for this study, and analyzed together with four published mtDNA sequences. The mtDNAs were within the sizes of 117 kb ~ 122 kb. The gene number was observed consistent except for two mtDNAs, which carry a duplicated *trnG1-trnG2* unit or a putative gene deletion. The size variation was largely attributed to the number of introns, repeated sequences, transposable elements (TEs), and plasmid-related sequences. Intron loss and gain were found from *cox1, rnl*, and *rns* of three mtDNAs. Loss of two introns in *cox1* of KY217797.1 reduced its size by 2.7 kb, making it the smallest *cox1* gene (8.4 kb) among the *cox1*s of the 25 mtDNAs, whereas gain of a Group II intron (2.65 kb) and loss of a Group I intron (1.7 kb) in *cox1* of MF774813.1 resulted in the longest *cox1* (12 kb). In *rnl* of *L. edodes*, we discovered four intron insertion consensus sequences which were unique to basidiomycetes but not ascomycetes. Differential incorporation of introns was the primary cause of the *rnl* size polymorphism. Homing endonucleases (HEGs) were suggestively involved in the mobilization of the introns because all of the introns have HEG genes of the LAGRIDADG or GIY-YIG families with the conserved HEG cleavage sites. TEs contributed to 11.04% of the mtDNA size in average, of which 7.08% was LTR-retrotransposon and 3.96% was DNA transposon, whereas the repeated sequences covered 4.6% of the mtDNA. The repeat numbers were variable in a strain-dependent manner. Both the TEs and repeated sequences were mostly found in the intronic and intergenic regions. Lastly, two major deletions were found in the plasmid-related sequence regions (*pol2*-*pol3* and *pol1*-*atp8*) in the five mtDNAs. Particularly, the 6.8 kb-long deletion at *pol2*-*pol3* region made MF774813.1 the shortest mtDNA of all. Our results demonstrate that mtDNA is a dynamic molecule that persistently evolves over a short period of time by insertion/deletion and repetition of DNA segments at the strain level.

## Introduction

Mitochondrion, the membrane-bound cellular organelle, is the powerhouse of eukaryotic cells that produces ATP through oxidative phosphorylation. It also involves in the cellular events like cell metabolism, calcium signaling, and apoptosis (Chan, 2006). Mitochondrion carries its own DNA (mtDNA), which is inherited independently of the nuclear DNA. The mitochondrial genome consists of genes for ATP generation, tRNAs, rRNAs, etc. The mtDNA size is highly dependent on organisms of which animals have relatively constant 16.4 kb mtDNAs whereas land plants have variable sizes with the average length of 412 kb (NCBI Genome^1^). The mtDNAs of the higher fungi, which include ascomycetes and basidiomycetes, have an average length of 65.2 kb and varies in size from 18.8 kb in *Hanseniaspora uvarum* (Pramateftaki et al., 2006) to 332 kb in *Golovinomyces cichoracearum* (Zaccaron and Stergiopoulos, 2021). Despite the size difference, the number of essential genes are relatively constant. The size diversity is mainly due to intron insertion, plasmid DNA insertion, partial duplication of mtDNA sequences, and changes in repetitive sequences (Hausner, 2003; Liu et al., 2020). The sequence variations make the mtDNA an active evolutionary genetic marker (Shen et al., 2015; Jelen et al., 2016; Robicheau et al., 2017; Zhang et al., 2017).

The mtDNA is acquired from one of the parent cells during the sexual reproduction(Sato and Sato, 2012). *Cryptococcus neoformans*, a basidiomycetous yeast, was observed to inherit the mtDNA in a uniparental way possibly through selective destruction of a mitochondrial type or through the emergence of bud where one parent is concentrated (Xu et al., 2000). *Ustilago maydis* also shows uniparental inheritance associated with a defined mating type, where the result is strongly affected by mating-type locus (Xu et al., 2000; Yan and Xu, 2003; Fedler et al., 2009). In the mating of filamentous basidiomyceteous fungi, two types of dikaryotic cells, which carry one of the two mtDNAs originated from the two monokaryotic cells, are generated through reciprocal entrance of nuclei to the cytoplasms of monokaryotic cells after hyphal fusion between monokaryotic hyphae with compatible mating types (Kim et al., 2019).

*Lentinula edodes*, shiitake mushroom, is a basidiomycete fungus that is a popular commercially cultivated mushroom. It is mainly found in East Asian countries, including China, Japan, and Korea (Menolli et al., 2022). Genomic DNA sequencing revealed variations in the genome size and gene number as the *L. edodes* B17 strain (Korea) consists of chromosomal DNA of 46.1 Mb with 13,028 predicted genes (Shim et al., 2016), while the *L. edodes* W1-26 strain (China) carries chromosomal DNA of 41.8 Mb with 14,889 predicted genes (Chen et al., 2016). The genetic diversity analysis using the mating type genes of 127 strains of *L. edodes* showed a high degree of diversity particularly in the *A* mating type locus of the wild strains (Ha et al., 2018). The diverse nature of the *A* mating type genes enabled the discrimination of nuclei in dikaryons as well as monokaryons of different origins (Kim et al., 2019). The mtDNA is another source of genetic information. Four mtDNAs of *L. edodes* have been reported thus far; they are AB697988.1, KY217797.1, MF774812.1, and MF774813.1 with the lengths of 121,394, 116,897, 119,134, and 115,116 bp, respectively. Gene annotation revealed the presence of 15 protein-coding genes (*cob*, *atp6, 8, 9*, *nad1-6, 4 l*, *cox1-3*, and *rps3*), two rRNAs (*rns* and *rnl*), 26 tRNAs, multiple intronic ORFs, and putative protein-coding genes (Song et al., 2019).

In recent years, we have analyzed the mtDNAs of different strains of *L. edodes* and found length polymorphism and the sequence variations in the mtDNAs of various origins, one of which was the presence of more than 25 variable-length tandem repeats in the intergenic and intronic regions, contributing the size polymorphism (Kim et al., 2019). For further understanding of the mtDNA diversification, we determined the mtDNA sequences in 20 selected strains from the 127 strains that were used for the *A* mating type analysis. This paper reports the detailed gene arrangement, variations in gene structure, and length polymorphism of the mtDNAs of *L. edodes*.

## Materials and methods

### Mushroom strains

The strains of *L. edodes* used for the mtDNA analysis were chosen on the basis of the *A* mating type diversity as verified in our previous paper (Table 1; Ha et al., 2018). The strains were maintained on a potato-dextrose agar (PDA). For the preparation of total DNA, the mycelia were cultured in potato-dextrose broth (PDB) for 10 days at 25°C. The mycelia were harvested by centrifugation for 10 min at 3,000 × g. The harvested mycelia were washed with a PBS buffer and then dried with a kitchen towel. Approximately 100 mg of dried mycelium was flash-frozen in liquid nitrogen, and then genomic DNA was extracted using the GenExTM Plant kit (GeneAll, Seoul, Korea). The extracted DNA was measured using a Micro-spectrophotometer K5600 (BioFuture Inc., China).

**Table 1:** Characteristics of mtDNAs of *Lentinula edodes* and some selected mushrooms.

Strains	Origin^a^	*A* mating type		mtDNA length (bp)	GC (%)	Sequence identity (%)^b^	Coverage (%)	Accession No.
Chunbeak_MT	KR	A5	A7	121,641	30.7	99.91	100	OP345457
Chunjang_MT	KR	A1	A5	121,504	30.7	99.91	100	OP345459
Baekwha_MT	KR	A4	A7	121,491	30.7	99.91	100	OP345454
Dasan_MT	KR	A1	A5	121,506	30.7	99.91	100	OP345455
SJ707_MT	KR	A5	A7	121,487	30.7	99.93	100	OP345463
Suhyang_MT	KR	A1	A7	121,489	30.7	99.93	100	OP345456
SJ701_MT	KR	A1	A5	121,489	30.7	99.93	100	OP345462
AB697988.1	JP	NA	NA	121,394	30.7	100	100	AB697988.1
SL7_MT	KR	A1	A15	121,487	30.7	99.93	100	OP328152
SJ301_MT	KR	A1	A15	121,486	30.7	99.92	100	OP345464
SJ302_MT	KR	A1	A7	121,486	30.7	99.92	100	OP345465
Gaeul_MT	KR	A11	A28	121,440	30.7	99.86	100	OP345453
Cham-B17_MT	KR	A1	A5	121,617	30.7	99.75	99	OP328157
L808_MT	CN	A1	A22	121,440	30.7	99.75	99	OP345461
Yeoreum_MT	KR	A18	A30	121,491	30.7	99.79	99	OP345452
Yujiro_MT	JP	A1	A5	119,233	30.8	99.67	98	OP328156
MF774812.1	CN	NA	NA	119,134	30.8	99.7	98	MF774812.1
SJ102_MT	KR	A17	A22	121,671	30.7	99.63	99	OP328158
SMR1_MT	KR	A1	A11	121,299	30.7	99.62	99	OP345460
KY217797.1	CN	NA	NA	116,897	30.7	98.7	96	KY217797.1
L54_MT	CN	A1	A2	119,500	30.8	99.5	98	OP328155
SL9_MT	KR	A27	A29	121,481	30.7	99.29	99	OP328153
SL10_MT	KR	A1	A31	119,219	30.8	99.62	99	OP328154
Pungnyun_MT	KR	A1	A7	121,508	30.7	99.57	99	OP345458
MF774813.1	CN	NA	NA	115,116	31.8	98.53	89	MF774813.1
*Moniliophthora roreri*				93,722	27.4	85.98	14	NC_015400.1
*Pleurotus ostreatus*				73,242	26.4	79.88	6	NC_009905
*Flammulina velutipes*				88,395	16.5	79.19	6	JF799107
*Agaricus bisporus* var. *bisporus H97*				135,005	35.9	90.1	4	JX271275

a Origin of strains: CN, China; JP, Japan; KR, Korea.

b Sequence identity was measured against AB697988.1 as a reference sequence.

### NGS reads, assembly, and phylogenetic analysis

The mtDNA sequences of the 21 selected *L. edodes* strains were assembled using the genomic DNA reads generated from the previous NGS sequencing (Shim et al., 2016; Lee et al., 2017). Briefly, the genomic DNA reads were mapped against AB697988.1 as a reference mtDNA by BWA (v.0.7.17; Li and Durbin, 2009). The mapped reads were assembled using SPAdes (v.3.12.0; Nurk et al., 2013), and the error-correction was performed using Pilon (v.1.22; Walker et al., 2014). The assembled mtDNA sequences were analyzed together with the four published *L. edodes* mtDNA sequences, including AB697988.1, KY217797.1, MF774812.1, and MF774813.1. MAFFT (v.7) online version^2^ was employed to compare the mtDNA sequences through multiple sequence alignment (Katoh et al., 2019). Phylogenetic tree was constructed using complete mtDNA sequences by the Neighbor-Joining method (Tajima-Nei model) with 1,000 times of Bootstrap resampling (Saitou and Nei, 1987).

### Mitochondrial gene annotation

The protein-coding genes, tRNAs, and introns in the mtDNA were annotated by MFannot and RNAweasel^3^ with manual corrections. The mtDNA sequences were deposited to GenBank with the accession numbers summarized in Table 1.

### Homing endonuclease and other protein analysis

The polypeptide sequence of homing endonucleases (HEGs) in the introns of mitochondrial genes were obtained through direct translation by Translate program^4^ using the genetic code of ‘Mold, protozoan and coelenterate mitochondrial, mycoplasma/spiroplasma’. Multiple sequence analysis on the HEGs was performed by Clustal Omega^5^ using default parameters. Unrooted tree was constructed by Neighbor-Joining method. InterPro at EBI^6^ was used for protein domain analysis. The transmembrane domain in a putative protein between *pol1* and *atp8* was predicted using Phobius program (Käll et al., 2007).

### Transposon and repeated sequence analyses

Transposons in the mtDNA of *L. eoddes* were analyzed using CENSOR server (Kohany et al., 2006) and GIRI database^7^. Tandem repeats were identified using Tandem Repeat Finder^8^ (Benson, 1999).

## Results

### Mitochondrial DNA diversity

The mtDNAs of 21 strains of *L. edodes* were assembled using genomic DNA reads. The sizes of the assembled mtDNAs were ranging from 119,219 bp to 121,671 bp (Table 1), which were similar to the previously published *L. edodes* mtDNA, such as AB697988.1 (121,394 bp), KY217797.1 (116,897 bp), MF774812.1 (119,134 bp), and MF774813.1 (115,116 bp; Yang et al., 2017; Song et al., 2019). The mtDNA of *L. edodes* was bigger than that of *Pleurotus ostreatus* (73,242 bp; Wang et al., 2008) and *Flammulina velutipes* (88,508 bp; Yoon et al., 2012), but smaller than that of *Agaricus bisporus* (135,005 bp; Férandon et al., 2013). SJ102_MT, which was the mtDNA from the SJ102 strain, containing rare composition of the *A* mating types (*A17* and *A22*), was the largest with the length of 121,671 bp. GC contents were mostly 30.7% while the mtDNAs of SL10, Yujiro, L54, and MF774812.1 showed slightly higher GC content (30.8%). Interestingly, MF774813.1, the smallest mtDNA in this study, had the highest GC content (31.8%). The mtDNAs of some other mushrooms, such as *A. bisporus*, *P. ostreatus, F. velutipes,* and *Moniliophthora roreri,* had GC contents of 35.9, 26.4, 16.5, and 27.6%, respectively.

**Figure 1:**
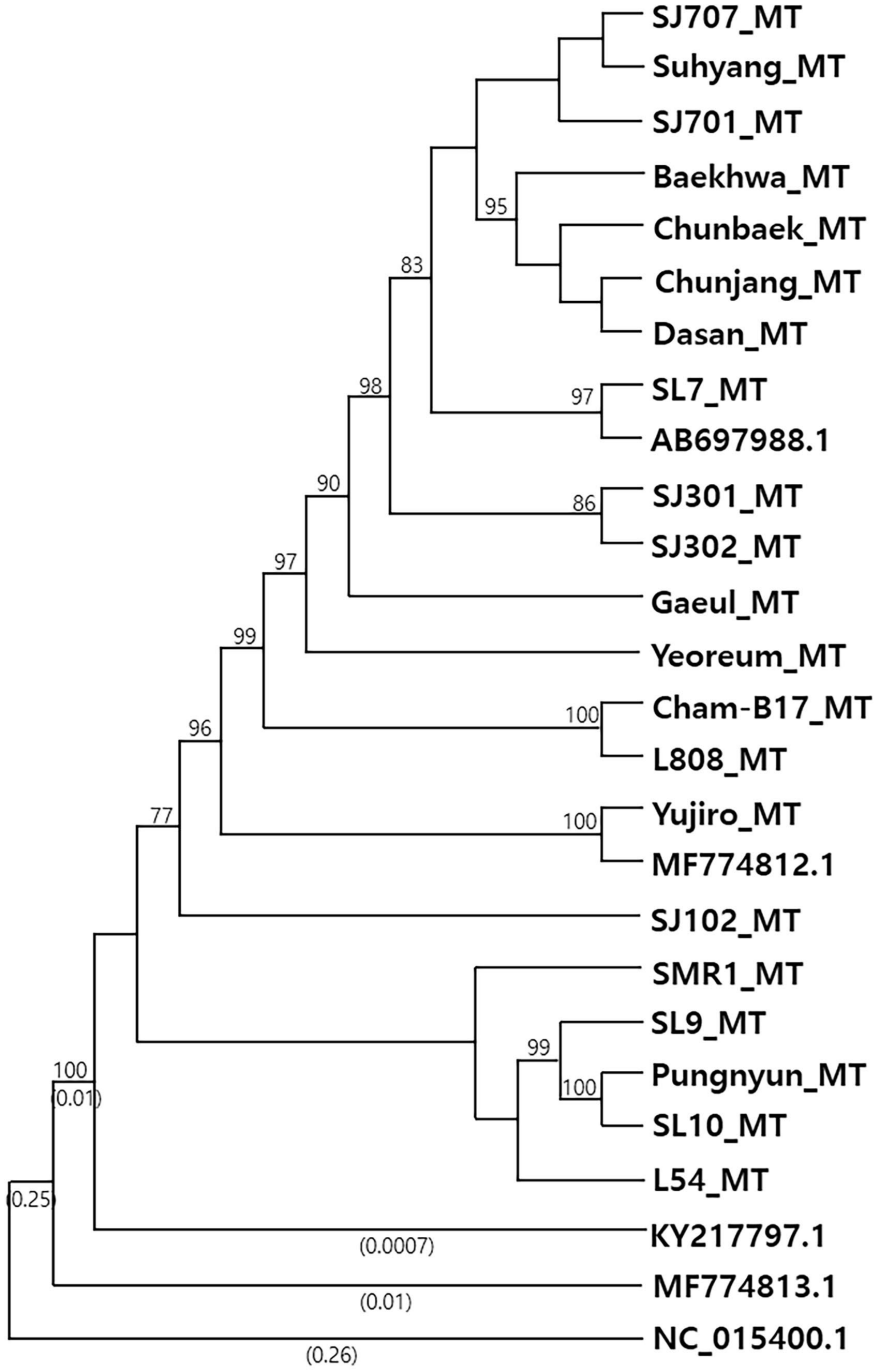
Phylogenetic analysis of *Lentinula edodes* mtDNAs. Phylogenetic tree was constructed using complete mtDNA sequences by the Neighbor-Joining method with 1,000 times of Bootstrap resampling. Bootstrap values greater than 70% are shown at branching points. Numbers enclosed in parentheses represent the branch lengths, and those shorter than 0.0005 are omitted. The mtDNA of *Moniliophthora roreri* (NC_015400.1) was used as an outgroup. Detailed information of the mtDNAs are summarized in Table 1.

Phylogenetic analysis of the mtDNA sequences revealed the diversification of *L. edodes* mtDNA (Figure 1). Nonetheless, all the sequences were highly homologous, showing near 99% identity each other (Table 1). MF774813.1 was the least related sequence, showing 98.53% sequence homology with AB697988.1. The mtDNAs of 10 cultivated strains, including SJ701, SJ707, SJ301, SJ302, Suhyang, Baekhwa, Chunbaek, Chunjang, Dasan, and SL7, were highly homologous together with AB697988.1. Sequence identity among these sequences is more than 99.9%. The strains with these mtDNAs carry nuclei with the *A* mating type pairs mainly consisted of *A1*, *A5*, and *A7*, which are the major *A* mating types found in the cultivated strains (Kim et al., 2019). This indicates that the cultivated strains have been bred within very narrow genetic pool. The mtDNAs found from the strains with rare *A* mating types, such as Gaeul (*A11*, A28), Yeoreum (*A18, A30*), SJ102 (*A17, A22*), SL9 (*A27, A29*), and SL10 (*A1, A31*), had more mtDNA sequence variations, showing 99.86%, 99.79%, 99.63%, 99.26%, and 99.62% sequence identity, respectively, with AB697988.1 (Table 1). There was a tendency that the strains with rare *A* mating types have the mtDNAs of higher sequence variations.

### Structure of the *Lentinula edodes* mtDNAs

**Figure 2:**
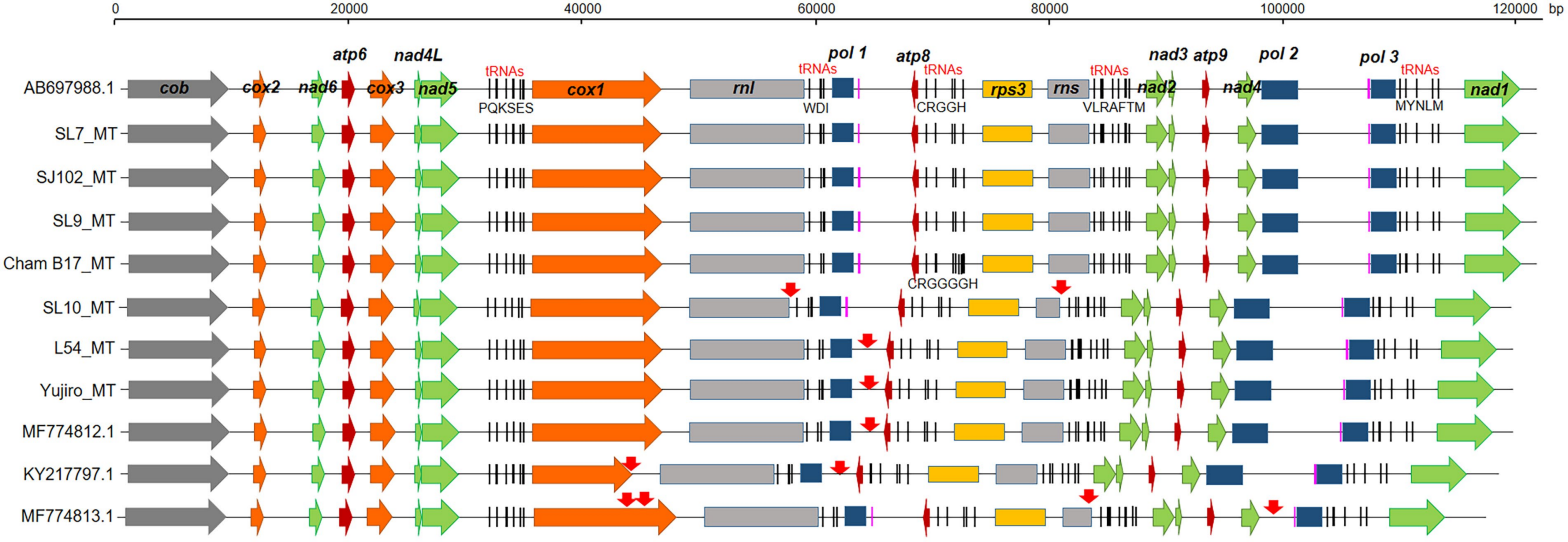
Gene arrangement of selected mtDNAs in different strains of *Lentinula edodes*. The mtDNA genes were annotated by MFannot. Regions of sequence variations are indicated by red arrows. Capital letters under the five tRNA regions indicate tRNAs having anticodons for corresponding amino acids. Two predicted tRNAs with unknown isotypes are colored in pink.

The *L. edodes* mtDNAs consisted of 15 protein-coding genes (*cob*, *atp6, 8, 9*, *nad1-6, 4 l*, *cox1-3*, and *rps3*), two rRNAs (*rns* and *rnl*), 26 tRNAs, and three putative DNA polymerase genes (Figure 2), showing the same result with previously published paper (Song et al., 2019). The only difference in the gene number of all the assembled mtDNAs was the presence two additional *trnG*s, tRNA genes having anticodon for glycine, in the mtDNA of Cham-B17 (Supplementary Figure S1). The four identical copies of *trnG*s with three single nucleotide polymorphisms (SNPs) were tandemly arranged and separated by the ATTTAA motif. The SNP analysis revealed that the *trnG1*-*trnG2* unit was duplicated in Cham-B17_MT (Supplementary Figure S1). The spatial arrangement of genes was essentially identical among the 25 mtDNAs but was highly different from that of *M. roreri* (NC_015400.1), a mushroom species belonged to Marasmiaceae family with the genus *Lentinula* (Supplementary Figure S2). Comparative analysis on the mtDNA structure revealed some major variations in the six mtDNA sequences (SL10_MT, L54_MT, Yujiro_MT, MF774812.1, MF774813.1, and KY217797.1; Figure 2, indicated by red arrows). The mtDNA of SL10 was shortened in *rnl* and *rns* by 954 bp and 1,353 bp, respectively. Deletion at the intergenic region (~2 kb) was observed in between *pol1* and *atp8* in the mtDNAs of L54, Yujiro, and MF774812.1. KY217797.1 was found to have two deleted regions, at the *cox1* gene and the intergenic region described above. MF774813.1 contained multiple variations. It has the same deletion at *rns* with SL10_MT. The *cox1* gene had an additional insertion which made it 1-kb larger than ordinary *cox1* found in other mtDNAs (See below). Moreover, MF774813.1 was found to have a large deletion (6.8 kb) in between *nad4* and *pol3* in which *pol2* was included. The *pol2* gene was assumed to be non-functional because its ORF was broken into two pieces.

### Intron mobilization in *cox1*

**Figure 3:**
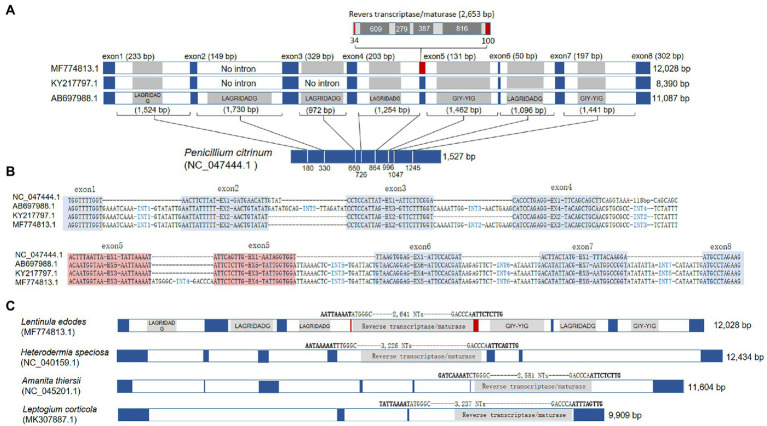
Gene structure variations of *cox1*. **(A)** Comparison of *Lentinula edodes cox1* (*Le*_*cox1*) with archetypal *cox1* in the mtDNA of *Penicillium citrinum (Pc_cox1)*. Intronic homing nuclease genes are shaded inside introns. Intron insertion sites at *Pc_cox1* are depicted by white lines with their positions from the 5′-end. The split exon by a group II intron in MF774813.1 is red-boxed. **(B)** Intron insertion sequences in *cox1*. Exonic regions are blue-shaded. The spilt exon in MF774813.1 are red-shaded. **(C)** The presence of homologous group II intron in different *cox1* of fungal mtDNAs. Exons are colored in blue. The intron insertion sequence and the length of the group II inton are provided at the top of the introns.

The major variations among the protein-coding genes were found in *cox1* of KY217797.1 and MF774813.1. The *cox1* gene of *L. edodes* mtDNA consisted of 8 exons separated by 7 introns (Figure 3A). All of the introns were group I introns, except for intron4 (group II; Supplementary Data S2) and have intronic ORFs encoding HEG of LAGRIDADG or GIY-YIG family. However, KY217797.1 lacked intron2 and intron3 which resulted in direct connection of exons 2–4 to a single exon. MF774813.1 was also devoid of intron2 but not intron3. Interestingly, MF774813.1 had a new intron insertion (2,653 bp) in exon5, dividing the exon5 (131 bp) into two exons of 34 bp and 100 bp. The inserted intron was a group II intron containing intronic ORFs homologous to reverse transcriptase/maturase found in *Amanita thiersii* (Li et al., 2020).

The intron incorporation in the *cox1* gene appears to occur independently. BLAST search with the exon sequence of *L. edodes cox1* found intronless *cox1*s from various fungal mtDNAs, such as *cox1* in the mtDNA of *Penicillium citrinum* (NC_047444.1) and *P. chrysogenum* (AM920464.1). The lengths of *cox1* in these organisms are 1,527 bp (*P. citrinum*) and 1,608 bp (*P. chrysogenum*) which are close to the length of *cox1* mRNA (1,602 bp) after removal of introns from 11 kb-long *cox1* gene in *L. edodes* mtDNA. Comparative sequence analysis of the *cox1* genes in *L. edodes* mtDNAs with that in *P. citrinum* revealed that each intron was inserted into a specific site possibly recognized by an HEG encoded by the intron (Figure 3A). For example, the first intron in *cox1* of *L. edodes* was inserted into an exonic consensus sequence composed of AGGTTTTGGT and AATTATTTTT, which was conserved in *P. citrinum* as TGGTTTTGGT and AACTTCTTAT (Figure 3B). Similar insertion to a specific consensus sequence was also observed in the introns 2–7 (Figure 3B). Loss of introns in KY217797.1 and MF774813.1 resulted in the connection of the consensus sequences thereby connecting exons. These consensus sequences may serve for recognition and cleavage sites for intronic HEGs (summarized in Supplementary Table S1; Megarioti and Kouvelis, 2020). Exon4 in MF774813.1 (corresponding to exon5 in AB697988.1) was particularly of interest since it was divided by a unique intron, not observed in other mtDNAs of *L. edodes*. BLAST analysis using the intron sequence found that the homologous sequences were present in the mtDNA *cox1* gene of *A. thiersii*, *Heterodermia speciosa*, and *Leptogium corticola*. All share a consensus sequence of AAAAT-ATT(C/T)(T/A) and encode a reverse transcriptase/maturase (Figure 3C).

### Variations in *rnl*

**Figure 4:**
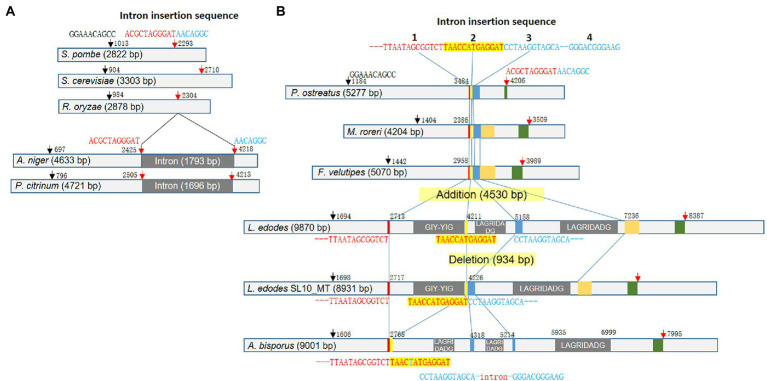
Variations of *rnl* in fungal mtDNAs. **(A)** Diversification of *rnl* in ascomycetes. The consensus sequences and the positions are depicted at the top of the gene scheme with arrows. Intron insertion splits the second consensus sequence into two pieces. **(B)** Diversification of *rnl* in basidiomycetes by intron insertion. The conserved sequences regions are color-boxed. The consensus sequences found in ascomycetes are also present in basidiomycetes (arrows). rnl in the mtDNA of *L. edodes* generally contains three introns with intronic HEG gene. However, SL10_MT lacked the second intron, resulting in the direct connection of the second and the third intron insertion sequences. *Rnl* of *A. bisporus* mtDNA has a new intron in between the third and the fourth intron insertion sequence.

*Lentinula edodes* mtDNA had a large *rnl* which encodes rRNA for ribosomal large subunit (LSU). *rnl* of *L. edodes*, 9,870 bp, was exceptionally large among LSU rRNAs of fungal mtDNAs (Figure 4). Fungi have *rnl* with the sizes ranging from 2.8 Kb to 5.3 Kb: 2,822 bp for *Schizosaccharomyces pombe* (NC_001326.1), 3,303 bp for *Saccharomyces cerevisiae* (NC_027264.1), 2,878 bp for *Rhizopus oryzae* (AY863212.1), 4,204 bp for *Moniliophthora roreri* (HQ259115.1), 5,070 bp for *Flammulina velutipes* (NC_021373.1), and 5,277 bp for *Pleurotus ostreatus* (NC_009905.1). Sequence analysis revealed that *rnl* of *L. edodes* contained three genes for intronic HEGs with the sizes of 1,498 bp, 947 bp, and 2,078 bp (Figure 4B). Each HEG gene encodes homing nuclease of GIY-YIG or LAGRIDADG. *rnl* of SL10_MT was the only mtDNA lacking the intronic region (934 bp) which contains the second LAGRIDADG HEG gene.

For better understanding of the *rnl* size polymorphism, we performed detailed sequence analysis using *rnl* sequences in mtDNAs of dikaryotic fungi. Firstly, we found that the fungi have common signature sequences which include GGAAACAGCC at the 5′-region and ACGCTAGGGAT-AACAGGC at the 3′-region (Figure 4A). In ascomycetes, the latter sequence serves as the site of intron incorporation (ACGCTAGGGAT-intron-AACAGGC), resulting in increase of the size of *rnl* by the intron insertion (~1.7 kb) as shown in *rnl* of *Aspergillus niger* (NC_007445.1, 4,633 bp) and *Penicillium citrinum* (NC_047444.1, 5,277 bp; Figure 4A). However, basidiomycetes were found to use other signature sequences for the intron incorporation although the ACGCTAGGGAT-AACAGGC signature was highly conserved across fungal groups. Instead, basidiomycetes have a characteristic sequence less than 1 kb before ACGCTAGGGAT-AACAGGC that is a sequential combination of TTAATAGCGGTCT, TAACCATGAGGAT, and CCTAAGGTAGCA-115-NTs-GGGACGGGAAG (Figure 4B). These sequences were found as a connected sequence in basidiomycetes of intronless *rnl*s such as *P. ostreatus* (5,277 bp), *Moniliophthora rorei* (4,204 bp), and *F. velutipes* (5,070 bp). However, these sequences became the signatures of intron insertion in the intron-containing *rnl* of mtDNAs. In *L. edodes*, the three sequences were separated by three units of introns, each of which encodes its own homing nuclease (Figure 4B). All the *rnl*s in mtDNAs of *L. edodes* in this study, except for SL10_MT, had the triple incorporation of introns with the total size of 4,530 bp, resulting in increase of *rnl* to the size of 9,870 bp. This mobilization of intron in *rnl* through the signature sequences was further supported by the deletion of the second intron in SL10_MT, leaving TAACCATGAGGAT and CCTAAGGTAGCA-115-NTs-GGGACGGGAAG as a connected sequence. It was also supported by the mode of intron insertion in *rnl* of mtDNA in *A. bisporus*. The *rnl* in *A. bisporus* lacked the first intron encoding GIY-YIG homing nuclease, leaving TTAATAGCGGTCT and TAACTATGAGGAT as a connected sequence, while maintaining the second and the third introns in between TAACTATGAGGAT and CCTAAGGTAGCA and after GGGACGGGAAG (Figure 4B). One interesting finding here was the presence of an additional intron found inside the CCTAAGGTAGCA-115-NTs-GGGACGGGAAG signature in which the intron replaced the 115-bp internal sequence. It contains a truncated gene encoding the C-terminus of LAGRIDADG homing endonuclease. Therefore, it was suggested that basidiomycetes use the three consensus sequences as the sites of intron incorporation for the diversification of *rnl* in the mtDNA while ascomycetes use a single consensus sequence, also conserved in but not used by basidiomycetes.

### Variation in *rns* and intergenic deletions

**Figure 5:**
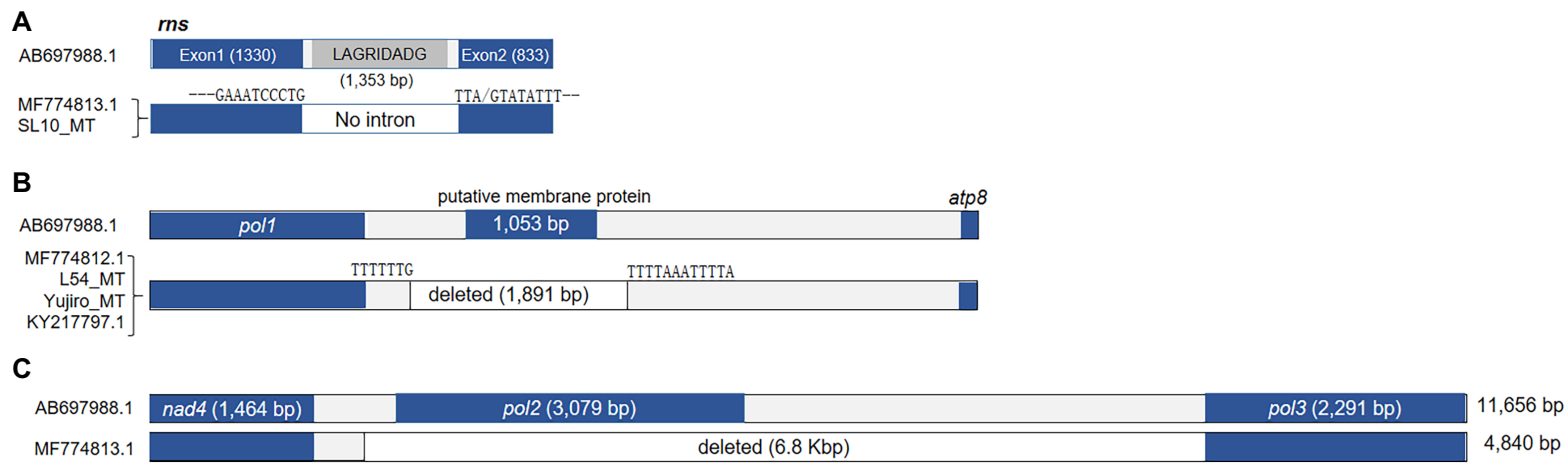
Sequence variations in *rns* and other deletions in the mtDNA. **(A)** The occurrence of intronless *rns* in MF774813.1 and SL10_MT. **(B)** Deletion of sequence region found in four mtDNAs. The deleted region contains a putative protein-coding gene. Domain analysis failed to find any conserved domain but the putative protein was predicted to be a membrane protein with 5 transmembrane domains. **(C)** A large deletion found from MF774813.1.

The *rns* gene of *L. edodes* mtDNA, encoding rRNA for small subunit of ribosome (SSU), was also larger than that of other fungi, due to the intron incorporation dividing *rns* into two fragments (1,330 and 833 bp; Figure 5A). The 1,353 bp-long intron encoded a LAGRIDADG HEG. SL10_MT and MF774813.1 were the two mtDNAs lacking this intron among the 25 *l. edodes* mtDNAs. The consensus sequence at the intron insertion site was GAAATCCCTG-TTA(G)TATATTT (Supplementary Table S1).

Some mtDNAs, including MF774812.1, L54_MT, Yujiro_MT, and KY217797.1, lacked 1.9 kb DNA region located in between *pol1* and *atp8* (Figure 5B). The lacking region contained a gene (1,053 bp) which encodes a putative protein (350 aa) with 5 transmembrane domains. Lastly, there was a large deletion (6.8 kb) in between *nad4* and *pol3* in MF774813.1 (Figure 5C). Because of this deletion, MF774813.1 becomes the smallest among all *L. edodes* mtDNAs in spite of the large insertion at *cox1* (Figure 3). *pol2* was the only gene included in this region. BLAST analysis of the deleted *pol2*-encoding protein revealed that it was homologous to RNA polymerase of *P. ostreatus* linear mitochondrial plasmid mlp2 while *pol3* was similar to DNA pol of *M. roreri* mitochondrial plasmid pMR3. Further phylogenetic analysis of *pol1-pol3* showed that they were related to the mitochondrial plasmid DNAs of fungi, such as pPE1A of *Pichia etchellsii* and pHC2 of *Hebeloma circinans* (Supplementary Figure S3).

### Homing endonucleases in *Lentinula edodes* mtDNAs

**Figure 6:**
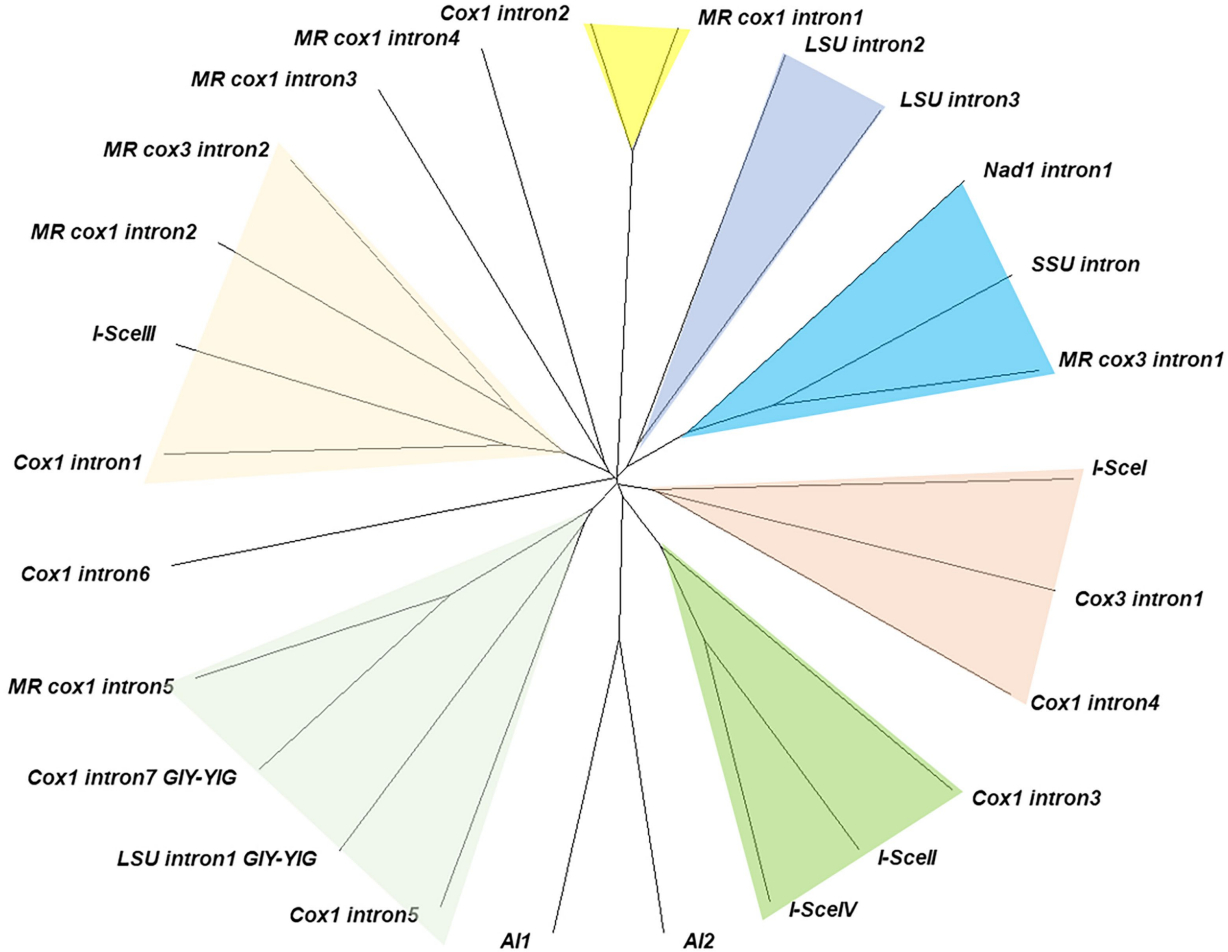
Grouping of intronic HEGs. Similar HEG groups are color-shaded. HEGs from introns in the genes of *M. roreri* are described as ‘*MR gene name intron*’ while HEGs of *Saccharomyces cerevisiae* are described following HEG naming rule. The unrooted tree was constructed using translated protein sequences by Neighbor-Joining method.

Thirteen homing endonuclease genes (HEGs) were discovered from the introns within *cox1* (7), *cox3* (1), *nad1* (1), LSU rRNA (3), and SSU rRNA (1). Ten of them were LAGRIDADG domain containing HEGs while the remaining three, including intronic ORFs found in intron5 of *cox1*, intron1 of LSU, and intron7 of *cox1*, were GIY-YIG HEGs. Comparative sequence analysis with six *S. cerevisiae* HEGs, including *I-SceI-IV*, *AI1* and *AI2*, and seven *M. roreri* HEGs, including MR_*cox1* intron1-5, and MR_*cox3* intron1 and intron2, showed that the HEGs were distributed in seven subgroups (Figure 6). The two LAGRIDADG domain-containing HEGs from intron 4 of *cox1*, intron 1 of *cox3*, which share significant homology with yeast mitochondrial *I-SceI*, forms a distinct group. The two LAGRIDADG domains were separated by 101 amino acid (aa) residues for both HEGs while 92 aa residues for *I-SceI* (Table 2). The yeast *I-SceII* (556 aa) and *I-SceIV* (630 aa) are long mtDNA intronic HEGs, which share highly homologous N-termini while the LAGRIDADG-containing C-termini are heterogeneous (Supplementary Data S1). The C-term of *I-SceII* was rather similar to HEG from intron3 of *cox1*. Both have two LAGRIDADG domains with the sequences of LAGLIDGDG and FS(V)GFFDADG separated by 97 aa residues. HEGs encoded by intron1 of *nad1*, intron of SSU, and intron1 of MR_*cox3* formed a distinct group (Figure 6). HEGs in this group contained heterogeneous LAGRIDADG domains: VTGFFDAEA and ISGFVSGEG for SSU intron, WTGLIDGEG and IAGFVTGEG for MR_*cox3* intron1, and VIGFSYAEV for *nad1* intron1 (Table 2). Interestingly, HEG of *nad1* lacked the second LAGRIDADG domain that makes this HEG as one of the two single LAGRIDADG domain-containing HEG among all *L. edodes* mitochondrial LAGRIDADG HEGs. HEG encoded by intron1 of *cox1* shared high homology with *I-SceIII*, MR_*cox1*_intron2, and MR_cox3_intron2 (Supplementary Data S1). HEGs in intron2 and intron3 of LSU showed significant homology around the LAGRIDADG-domain, however, the former was a short HEG (196 aa) with a single LAGRIDADG whereas the latter was a long HEG (365 aa) with two LAGRIDADG-domains (Supplementary Data S1). HEG in intron2 of *cox1* was highly homologous to HEG in intron1 of MR_*cox1*. There was three GIY-YIG HEGs in the mtDNA of *L. edodes*, which were found from intron5 and intron7 of *cox1*, and intron1 of LSU. Homologous protein was also found from intron5 of MR_*cox1*, which showed better homology with HEG from intron7 of *cox1* than that from intron 5 of *cox1* (Supplementary Data S1).

**Table 2:** Homing endonucleases in *L. edodes* mtDNAs.

Species	Gene	HEG intron	HEG family	Length (aa)	Domain1	Domain2	Distance between domains (bp)
*L. edodes*	*cox1*	intron1	2LAGRIDADG	319	LAGLIEADG	LAGFTDGDG	110
		intron2	2LAGRIDADG	440	LVGVTDGDG	LVGFTEAEG	143
		intron3	2LAGRIDADG	288	LAGLIDGDG	FSGFFDADG	97
		intron4	2LAGRIDADG	214	IIGLILSDA	LAHWILGDG	100
		intron5	GIY-YIG	367	GVY	YIG	10
		intron6	2LAGRIDADG	255	WVGLMDAEG	LSGFVEAEA	129
		intron7	GIY-YIG	289	IIY	YVG	10
	*cox3*	intron1	2LAGRIDADG	223	IIGSLLGDG	LAVWMMDDG	100
	*nad1*	intron1	LAGRIDADG	184	VIGFSYAEV	-	
	*rnl*	intron1	GIY-YIG	324	FVY	YLG	10
		intron2	LAGRIDADG	196	LGGFVEGEG	-	
		intron3	2LAGRIDADG	367	VTGLTDGEG	VQTYLTGES	172
	*rns*	intron1	2LAGRIDADG	401	VTGFFDAEA	ISGFVSGEG	151
*Saccharomyces cerevisiae*		I-SceI	2LAGRIDADG	235	GIGLILGDA	LAYWFMDDG	92
		I-SceII	2LAGRIDADG	556	LAGLIDGDG	FVGFFDADG	97
		I-SceIII	2LAGRIDADG	321	LAGLIEGDG	LAGMTDADG	123
		I-SceIV	2LAGRIDADG	630	MTGILLTDG	LAHMIMCDG	101
*Moniliophthora rorei*	*cox1*	intron1	2LAGRIDADG	413	LVGVTDGDG	LVGFTEAEG	141
		intron2	2LAGRIDADG	357	LTGLIEGDG	LSGIIESDG	113
		intron3	2LAGRIDADG	297	LIGFSEGEG	ISGITDGEG	110
		intron4	2LAGRIDADG	298	LAGLIEGDG	LAGFTQADG	96
		intron5	GIY-YIG	242	IIY	YVG	10
	*cox3*	intron1	2LAGRIDADG	373	WTGLIDGEG	IAGFVTGEG	150
		intron2	2LAGRIDADG	395	LAGLLEGDG	LAGLIDSDG	111

### Transposons and repeated sequences in the mtDNA

**Figure 7:**
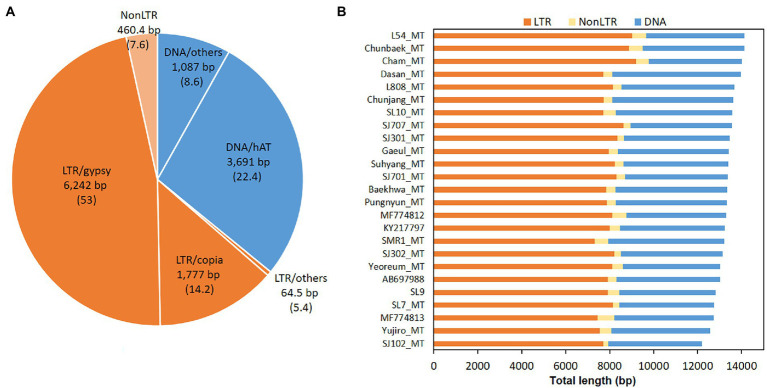
Transposable elements (TEs) in the mtDNA of *L. edodes*. TEs in 25 mtDNAs were predicted by Censor program. **(A)** Average contents of TEs. Retrotransposons are orange-colored while DNA transposons are in blue. Average number of each TE is described in parenthesis. **(B)** Total length of TEs in different mtDNAs.

**Figure 8:**
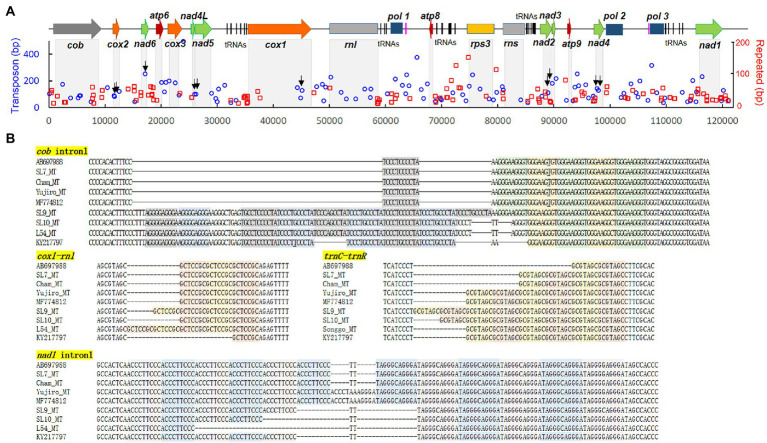
Repeated sequences in the mtDNA of *L. edodes*. **(A)** Locations of the repeated sequences (red rectangle) together with TEs (blue circle) along with the mtDNA sequence. The axis depicts the length of each TE or repeated sequence. Arrows indicate the positions of TEs in exons. **(B)** Sequence structure of repeated sequences at various locations in the mtDNA of different strains. The repeating units are colored differently.

Transposable elements (TEs) are some of the major factors in the genome modification. Computational analysis predicted that the *L. edodes* mtDNA contained 106.3 TE-related sequences in average (Figure 7A). However, there was no intact form remained. All of them were fragments of TEs with the sizes ranging from 50 bp to 250 bp. Sum of the total TE fragments was 13,322 bp per 120,700 bp mtDNA which covered 11.04% of the mitogenome (Figure 7A; Supplementary Table S2). LTR retrotransposon and DNA transposon were the major TEs with the composition of 7.08% (8,083 bp) and 3.96% (4,778 bp), respectively, while NonLTR retrotransposon (460 bp) was a minor TE family (Figure 7B). In LTR retrotransposon, LTR/gypsy and LTR/copia were most of the retrotransposons, covering 6,242 bp (53 count) and 1,777 bp (14.2 count), respectively, of the total retrotransposon content (8,543 bp; Figure 7A; Supplementary Table S3). Various DNA transposons, such as hAT, ExSpm/CACTA, and Mariner, were discovered with hAT as the sole major DNA transposon. 22.4 copies of hATs with the total length of 3,691 bp were scattered in different locations in the mtDNA (Figure 7A; Supplementary Table S3). TEs were largely located intronic and intergenic regions (Figure 8A, blue color). Ten of the 106.3 REs were found in the exonic regions of the protein-coding genes, including *cox2* (2), *nad6* (1), *nad5* (2), *cox1* (1), *nad2* (2), and *nad4* (2) (Figure 8A, black arrows). Of the protein-coding genes, *cob*, *cox3*, *cox1*, and *nad1*were found to carry TEs only in the intronic regions. *Rnl* had 7 TEs whereas none was included in *rns*.

Repeated sequences are frequently occurring DNA sequences in the genomes of living organisms. In our RepeatFinder analysis using AB697988.1, *L. edodes* mtDNA was found to contain 115 repeat sequences with the total length of 5,641 bp (4.6% of total mitogenome; Supplementary Data S2). Average length and copy number of the repeating unit were 12.7 bp and 5.2 copies. All of the repeats were found either inside the intron or in the intergenic region. In the intronic regions of protein-coding genes, 7 repeated sequences were found from *nad1*and *cob*, and 2 from *cox1* and *cox3* (Figure 8A; Supplementary Data S2). Some of the most frequent repeated sequences were ACCCTTCCC, TAGGGCAGGGA, GCTCCGC, TCCCTCCCCTA, and GGGAAGGGT as we previously reported (Kim et al., 2019). There were two types of repeats: one is the symmetrical repeats paring ACCCTTCCC with TAGGGCAGGGA occurred in intron1 of *nad1* and intron2 of *cob* and TCCCTCCCCTA with GGGAAGGGT in intron1 of *cob* and intergenic region between *trnC* and *trnR*, another is direct repeats of GCTCCGC (*nad1* intron1, *nad6-atp6*, and *trnY-trnN*), GCGTAGC (*trnC-trnR*), TATAA (*atp9-nad4*), etc. (Figure 8B). The repeating units occurred in different copy numbers in different mtDNAs. For example, a single copy of TCCCTCCCCTA was present in AB697988, SL7_MT, Cham-B17_MT, Yujiro_MT, and MF774812 while SL10_MT, L54_MT, and KY217797 had 5 copies and SL9_MT had 6 copies. GGGAAGGGT units occurred in 4–5 copies in opposite sides (Figure 8B).

## Discussion

In most eukaryotic species, the mitochondria are crucial cellular organelles in charge of energy metabolism. They have mtDNAs with a variety of sizes that contain their own genetic information, depending on the type of organism. The mtDNA size in higher fungi, which include ascomycetes and basidiomycetes, varies from 18.8 kb in the yeast *H. uvarum* (Pramateftaki et al., 2006) to 332 kb in the plant pathogen *G. cichoracearum* (Zaccaron and Stergiopoulos, 2021). The huge mtDNA of *G. cichoracearum* comprises extraordinarily large quantities of intron (207 kb, 62% of total mtDNA) and intergenic regions (104 kb, 31%), whereas the mtDNA of *H. uvarum* has primarily intronless genes and short intergenic sequences, suggesting that lateral gene transfer plays a great role in the evolution of mitochondria in fungal species.

In this study, we assessed the strain-level mtDNA variations using the mtDNA sequences of 25 different strains of *L. edodes* whose sizes range from 115,116 bp to 121,671 bp. The mtDNAs are homologous to each other with high sequence identity (Table 1). Particularly, the mtDNAs from the strains having frequently found *A* mating type pairs, consisted of *A1*, *A5*, and *A7* (Kim et al., 2019), are essentially identical by sharing more than 99.9% sequence identity. The mtDNAs from the strains with the uncommon *A* mating types, however, show larger sequence variations (Table 1). The tendency found here shows that very few nuclear and mitochondrial genetic pools have been used to breed the cultivated strains.

The total number of mitochondrial genes within the 25 mtDNAs of *L. edodes* was constant, except for two mtDNAs. Cham-B17_MT carries two extra copies of homologous *trnG* separated by ATTTAA motifs, totaling four tandem *trnG*s, through duplication of *trnG1*-*trnG2* unit (Figure 2; Supplementary Figure S1). The duplication found here could arise from replication slippage as discussed by Moritz and Brown (1987) using the ATTTAA motif as the repeating unit. Similar duplication of two tRNA unit has been found from the mtDNAs of *Tagiades vajuna* (Liu et al., 2017) and *Odontoptilum angulatum* (Liu et al., 2021). Another gene number variation was found from MF774813.1 in which the *pol2* gene was deleted together with a large non-coding region (Figure 5C). The *pol2* gene product is homologous to DNA polymerase type B (YP_009710628) of *Amanita brunnescens*, and is thought to be non-functional since it is broken. Since mtDNA replication depends on the activity of a nucleus-encoded DNA polymerase γ (*MIP1*; Foury, 1989), the true physiological function of the *pol* genes (*pol1*-*pol3*) is unknown. They can be remnants of mitochondrial plasmid DNAs which had been integrated into mtDNA and modified during further evolution (Weber et al., 1995; Wang et al., 2008; Férandon et al., 2013). Given the fact that the deletion only occurred in MF774813.1, the effect of the *pol2* loss there deserves further investigation. Additionally, there was deletion of a putative gene in the intergenic region between *pol1* and *atp8* which was predicted to encode a 350 aa-membrane protein (Figure 5B). It appears to be nonessential since several mtDNAs are devoid of this sequence.

Some of the mitochondrial genes, including *cox1*, *rnl*, and *rns*, showed size variation that was caused by intron mobilization. For example, we found that *L. edodes cox1* (Le_*cox1*) has been expanded to 11,087 bp by seven independent events of Group I intron insertion by the sequence comparison with an archetypal intronless *cox1* from *P. citrinum* (1, 527 bp; Figure 3). Each intron insertion site retains its own consensus sequence, presumably recognized by an intronic HEG. In fact, most of the consensus sequences discovered here are homologous to the insertion sequences of HEGs in fungal mtDNAs described by Megarioti and Kouvelis (2020). The size variations in Le_*cox1* were found from KY217797.1 and MF774813.1; the former has a variant Le_*cox1* with a size of 8,390 bp, and the latter with a size of 12,028 bp. The size reduction in Le_*cox1* of KY217797.1 is attributed to the absence of intron2 (1,720 bp) and intron3 (972 bp), resulting in the smallest Le_*cox1*. Providing the fact that KY217797.1 is more ancient mtDNA as shown by our phylogenetic analysis (Figure 1), Le_*cox1* in KY217797.1 is conceivably an earlier version in which the intron insertion has not yet taken place. This is further corroborated by the finding of Le_*cox1* in MF774813.1, which has intron3 but not intron2. Sequential insertion of intron3 and intron2 may occur to generate Le_*cox1* of full size. Le_*cox1* of MF774813.1 has another intriguing characteristic in that it evolves into a new form of *cox1* by incorporating a new group II intron (2,653 bp) into exon4. The new intron is the only Group II intron discovered from the mtDNA of *L. edodes,* and because of its rarity, it is thought to have been recently acquired. Homologs have been discovered from *cox1* of *A. thiersii*, *Heterodermia speciosa*, and *Leptogium corticola* (Figure 3C) with a distinct intron insertion sequence homologous to the exon binding sequence in *cox1* of *S. cerevisiae* (Eskes et al., 2000; Lambowitz and Zimmerly, 2004), indicating lateral gene transfer may have mobilized this retroelement beyond the boundaries of species. The mtDNA of *L. edodes* has relatively large *rnl* (9,870 bp) for mushrooms when compared with *rnl*s in *P. ostreatus* (5,277 bp), *M. roreri* (4,204 bp), and *F. velutipes* (5,070 bp; Figure 4). The size expansion here is attributed to the presence of three Group I introns, totaling 4,530 bp. Removal of them can result in *rnl* of the size close to above mentioned mushrooms. Investigation of the intron insertion sites reveals a serial connection of consensus sequence units composed of TTAATAGCGGTCT, TAACCATGAGGAT, CCTAAGGTAGCA, and GGGACGGGAAG through which mushrooms can incorporate at least three introns (Chen et al., 2021). In contrast to mushrooms, *rnl*s of ascomycetes in this study have different consensus sequences which are composed of ACGCTAGGGAT and AACAGGC. A single intron with the size of 1.7 kb has been inserted in between these sequences. One noteworthy feature of these sequences is that they are also present in basidiomycetes but are never used for intron insertion.

Together with gene number variation and intron mobilization, transposable elements (TEs) and repeated sequences (RSs) also contributed to the mtDNA size variations in *L. edodes*. TEs and RSs cover 11.04 and 4.6% of the mtDNA, respectively, mostly locating intronic and intergenic regions. TEs in this study are predicted to be inactive since all of them are fragments of TEs. They may have originated from the nuclear genome (Farrelly and Butow, 1983; Alverson et al., 2010), but more research is need to verify their exact origin and significance in the evolution of mitochondria. RSs appear to occur in a random manner since different numbers of RSs have been detected even in the closely related mtDNAs (Figure 8B). They can occur during DNA replication through polymerase slippage and possibly in the DNA repair process (Bzymek and Lovett, 2001; Phadnis et al., 2005). Lastly, plasmid insertion is one of the factors causing mtDNA size expansion (Sederoff, 1984; Férandon et al., 2013; Mardanov et al., 2014). The mtDNA of *Agaricus bisporus* was reported to contain plasmid-related sequences in two separated regions, covering 5% of mtDNA size (Férandon et al., 2013). Genes found in these regions were DNA polymerase and RNA polymerase genes, which originated from pEM plasmid. Similarly, the mtDNA of *Sclerotinia borealis* has plasmid-related DNA pol and RNA pol genes with additional related remnant sequences (Mardanov et al., 2014). The mtDNA of *L. edodes* in this study also carries two complete *pol* genes and an incomplete one in two separated regions. Considering the region between *pol2-pol3* and *pol1-atp8* as plasmid-related sequences, they can contribute more than 10% of the total mtDNA in *L. edodes*.

In conclusion, our research demonstrates that replication errors, such as gene duplication and RS generation, and the mobilization of DNA fragments, including introns, TEs, and plasmids, are the primary factors that allow persistent evolution of mtDNA even within a species.

## Data availability statement

The datasets presented in this study can be found in online repositories. The names of the repository/repositories and accession number (s) can be found at: https://www.ncbi.nlm.nih.gov/genbank/, OP328153; https://www.ncbi.nlm.nih.gov/genbank/, OP328154; https://www.ncbi.nlm.nih.gov/genbank/, OP328155; https://www.ncbi.nlm.nih.gov/genbank/, OP345458; https://www.ncbi.nlm.nih.gov/genbank/, OP345460; https://www.ncbi.nlm.nih.gov/genbank/, OP328158; https://www.ncbi.nlm.nih.gov/genbank/, OP328156; https://www.ncbi.nlm.nih.gov/genbank/, OP345452; https://www.ncbi.nlm.nih.gov/genbank/, OP345461; https://www.ncbi.nlm.nih.gov/genbank/, OP328157; https://www.ncbi.nlm.nih.gov/genbank/, OP345453; https://www.ncbi.nlm.nih.gov/genbank/, OP345465; https://www.ncbi.nlm.nih.gov/genbank/, OP345464; https://www.ncbi.nlm.nih.gov/genbank/, OP328152; https://www.ncbi.nlm.nih.gov/genbank/, OP345462; https://www.ncbi.nlm.nih.gov/genbank/, OP345456; https://www.ncbi.nlm.nih.gov/genbank/, OP345463; https://www.ncbi.nlm.nih.gov/genbank/, OP345455; https://www.ncbi.nlm.nih.gov/genbank/, OP345454; https://www.ncbi.nlm.nih.gov/genbank/, OP345459; https://www.ncbi.nlm.nih.gov/genbank/, OP345457.

## Author contributions

SK and HE analyzed the mtDNA gene structure and intron sequences. RN did the mtDNA annotation and contributed to the manuscript writing. YC did the mtDNA annotation. HL and HR generated the NGS sequences and provided the assembled mtDNA sequences. H-SR leaded the whole project from conceptualization to manuscript writing. All authors contributed to the article and approved the submitted version.

## Funding

This work was supported by Golden Seed Project (Grant No: 213007–05-4-WTH21) from the Ministry of Agriculture, Food and Rural Affairs, Korea.

## Conflict of interest

The authors declare that the research was conducted in the absence of any commercial or financial relationships that could be construed as a potential conflict of interest.

## Publisher’s note

All claims expressed in this article are solely those of the authors and do not necessarily represent those of their affiliated organizations, or those of the publisher, the editors and the reviewers. Any product that may be evaluated in this article, or claim that may be made by its manufacturer, is not guaranteed or endorsed by the publisher.

## Supplementary material

The Supplementary material for this article can be found online at: https://www.frontiersin.org/articles/10.3389/fmicb.2022.1034387/full#supplementary-material

## Footnotes

^1^ https://www.ncbi.nlm.nih.gov/genome/organelle/

^2^ https://mafft.cbrc.jp/alignment/server/

^3^ https://megasun.bch.umontreal.ca/RNAweasel/

^4^ https://web.expasy.org/translate/

^5^ https://www.ebi.ac.uk/Tools/msa/clustalo/

^6^ https://www.ebi.ac.uk/interpro/

^7^ http://www.girinst.org/censor/index.php

^8^ http://tandem.bu.edu/trf/trf.html
