# Supplementary material for: Comparative structural analysis on the mitochondrial DNAs from various strains of Lentinula edodes
Source: Front Microbiol. 2022 Nov 28;13:1034387. doi: 10.3389/fmicb.2022.1034387 (PMC9744193; doi:10.3389/fmicb.2022.1034387)
Supplement: Supplementary file 1 [file Data_Sheet_1.DOCX]

**Supplementary data 1.** Sequence alignment of HEGs

1. cox1_intron3 HEG, I-SceII, and I-SceIV

cox1_intron3 ------------------------------------------------------------ 0

I-SceII MVQRWLYSTNAKDIAVLYFMLAIFSGMAGTAMSLIIRLELAAPGSQYLHGNSQLFNVLVV 60

I-SceIV MVQRWLYSTNAKDIAVLYFMLAIFSGMAGTAMSLIIRLELAAPGSQYLHGNSQLFNVLVV 60

cox1_intron3 ------------------------------------------------------------ 0

I-SceII GHAVLMIFFLVMPALIGGFGNYLLPLMIGATDTAFPRINNIAFWVLPMGLVCLVTSTLVE 120

I-SceIV GHAVLMIFFLVMPALIGGFGNYLLPLMIGATDTAFPRINNIAFWVLPMGLVCLVTSTLVE 120

cox1_intron3 ------------------------------------------------------------ 0

I-SceII SGAGTGWTVYPPLSSIQAHSGPSVDLAIFALHLTSISSLLGAINFIVTTLNMRTNGMTMH 180

I-SceIV SGAGTGWTVYPPLSSIQAHSGPSVDLAIFALHLTSISSLLGAINFIVTTLNMRTNGMTMH 180

cox1_intron3 ------------------------------------------------------------ 0

I-SceII KLPLFVWSIFITAFLLLLSLPVLSAGITMLLLDRNFNTSFFEVSGGGDPILYEHLFWFFG 240

I-SceIV KLPLFVWSIFITAFLLLLSLPVLSAGITMLLLDRNFNTSFFEVSGGGDPILYEHLFWFFG 240

cox1_intron3 ----------------------------------------------------------MN 2

I-SceII QTVATIIMLMMYNDMHFS-KCWKL--------------------LKKWITNIMSTLF--- 276

I-SceIV HPEVYILIIPGFGIISHVVSTYSKKPVFGEISMVYAMASIGLLGFLVWSHHMYIVGLDAD 300

cox1_intron3 TNNISL--NSILAL--------LVKDSFKRKWNQQVTNNRRNTSHLVGTSETTRA----T 48

I-SceII -KALFV------------KM----FMSYNNQQDKMMNNTMLKKDNIKRSSETTRKMLNNS 319

I-SceIV TRAYFTSATMIIAIPTGIKIFSWLMNPFSKDKNKNKNKKLI---------RNYQKMNNNN 351

. :... :: .:. .. : .

cox1_intron3 SFSIKENRFNQWLAGLIDGDGSFQVS---KAGYSSCEITVSLADERMLRIIQNKLGGSIK 105

I-SceII MNKKFNQ----WLAGLIDGDGYFGIV---SKKYVSLEITVALEDEMALKEIQNKFGGSIK 372

I-SceIV MMKTYLNNNNMIMMNMYKGNL-YDIYPRSNRNYIQP----------------------NN 388

. : : .: .*: : : . * . :

cox1_intron3 PRSGVKAIRWRLHNRSGMIDLVKRVNGYIRHSSRLVQLNKICTVLDLQLLSPDTLHKKHG 165

I-SceII LRSGVKAIRYRLTNKTGMIKLINAVNGNIRNTKRLVQFNKVCILLGIDFIYPIKLTKDNS 432

I-SceIV INKELVVYGYNLESCVGMPTYTNIVKHMVGIPN-----NILY------------------ 425

.. : . :.* . ** : *: : . * :

cox1_intron3 WFSGFFDADGTVEFYLKGNSNNPQLTLSVTNKLY-------------------------- 199

I-SceII WFVGFFDADGTINYSFKNNHPQ--LTISVTN----------------------------- 461

I-SceIV IMTGILLTDGWIDYTSKKDLDK--KTIMEINCRFRLKQSMIHSEYLMYVFMLLSHYCMSY 483

: *:: :** ::: * : : *: *

cox1_intron3 ---------------------------------------------VDI------VFF--- 205

I-SceII ------------------------------------------KYLQDVQEYK-------- 471

I-SceIV PKMKIAKVKGKSYNQLEFYTRSLPCFTILRYMFYNGRVKIVPNNLYDLLNYESLAHMIMC 543

*:

cox1_intron3 --YNCFGGQVYFDKSQNGYYKWSVQSELGLESFL-EYT-----KTCP-PQSVKRNRLFLI 256

I-SceII ---NILGGNIYFDKSQNGYYKWSIQSKDMVLNFINDYIKMNPSRTT------KMNKLYLS 522

I-SceIV DGSFVKGGGLYLNLQS-------FTTKEL--IFIMNILKIKFNLNCTLHKSRNKYTIYMR 594

** :*:: .. . :: *: : . : :::

cox1_intron3 KEYYRLTRLKAHKYPEGTILHKAWLNFNKKWN------- 288

I-SceII KEFYNLKELKAYNKSSDSMQYKAWLNFENKWKNK----- 556

I-SceIV VESVKRLFPMIYKYILPSMRYKFD---IMLWQKKYNMIN 630

* . :: :: :* *:

1. cox1_intron1 HEG and I-SceIII

I-SceIII MENKEYNLKLNYDKLGPYLAGLIEGDGSITVQNSSSMKKSKYRPLIVVVFKLEDLELANY 60

Cox1_intron1 ------------------------------------------------------------ 0

I-SceIII LCNLTKCGKVYKKINRNYVLWTIHDLKGVYTLLNIINGYMRTPKYEAFVRGAEFMNNYIN 120

Cox1_intron1 ---------------------------------------MRTPKIESLHRAIDWLNVHTN 21

***** *: *. :::* : *

I-SceIII STTITHNKLKNMDNIKIKPLDTSDIGSNAWLAGMTDADGNFSINLMNGKNRS----SRAM 176

Cox1_intron1 ------------ANLVKQDLDRSDIDSNSWLAGFTDGDGNFSINLVDRKKRGLITTKRVQ 69

*: : ** **:.**:* **:**.*****:* :: *:*. .*.

I-SceIII PYYCLELRQNYQKNSNNNNINFSYFYIMSAIATYFNVNLYSRERNLNLLVSTNNTYKTYY 236

Cox1_intron1 AFFRIELRQNYHREVLSKLEGVSYFYILNKIACYLGVNLYSRTRVKGE--------KIFH 121

:: :******::: .: ..*****:. :* *:.****** * . * ::

I-SceIII SYKVMVANTYKNIKVMEYFNKYSLLSSKHLDFLDWSKLVILINNEGQSMKTNGSCELGMN 296

Cox1_intron1 SFMLISHNINSHQKTIEYFEKYPLYSSKYFAYKDWLNVVSKIISRNGSPLSQEDVKEIEK 181

*: : * .: *.:***:** * ***:: : ** ::* * ... * :: . : :

I-SceIII LRKDYNKTRTTFTWSHLKNTYLENK 321

Cox1_intron1 IKSQFNSKRTLFDFTHLDTIL---- 202

::.::*..** * ::**..

1. HEGs in LSU_intron2 and intron3

LSU_intron3 --KSMKTKTIINKNITNNSNRYRFLGFAGVAYLNNIDFTVPRRFEKLKPWQVTGLTDGEG 58

LSU_intron2_ MTKINNNNNIINRDVQ----------------------------DQSYLFFLGGFVEGEG 32

* :.:.::*::: :: : : *:.:***

LSU_intron3 SFICTISDTGKGVTGKVVNLEFKVTQKSHSMGILYELQEFFNCGSVVIDNRETDTKKYRI 118

LSU_intron2_ SNSVSISINRNFKFGVNIQPVFNVSQHKNGLDILYSFKELFKSGSVVEKSGSPDIFVYTL 92

* :** . : * :: *:*:*:.:.:.: *. :* *:.**** .. . * * :

LSU_intron3 KSLESILEKIIPHFESYP----CLTSKYLNYRDWKKIALIMKNKEHLTIEGINKIIEISS 174

LSU_intron2_ KGYKQIIQHVLPFLETYVQPFSCKKEEFSIF---KQIVLDSSEGKQKDRE---NLIEMIK 146

*. :.::::::*. *:* * ..:: : *::.* .: :: * :::*: .

LSU_intron3 KMNKARSFEDKYNYCKTSLGM--TTAPSISSLDKIKVQQNSEGEGSTVEIKYNLPCHWVQ 232

LSU_intron2_ -------------LCYRLQGKGKNRKRKLSEVL---------------EIVENK-TIYFD 177

* * . .:*. *: * :.:

LSU_intron3 TYLTGESMFYTYLGEKKSRGIVYQGCDSSLELGQNSHDVAILLSLKKFFNGGYIKPKYNY 292

LSU_intron2_ NLIINKEIQLRDLETEDSE----------------------------------------- 196

. : .:.: * :.*.

LSU_intron3 DNLYECLNSRSLNRYILRNTETIIKFVDKYPMLTRKHLDYLDWKKIVELKSRGAHKTEEG 352

LSU_intron2_ ------------------------------------------------------------ 196

LSU_intron3 LALIKEIISKVNSGR 367

LSU_intron2_ --------------- 196

1. HEGs in nad1_intron1, SSU_intron

Nad1_intron1 EKLSNSGNALKLMIPSRNRKVICGQNNYLGMVTIYKMSENEMGNRGSKSIL--------- 51

MR_cox3_intron1 ------------------------------MVISQEMNENEMGYRGSKSVL--------- 21

SSU_intron -----------------------GWNNYSCMVISQKMSESEMGYRGSKSEFKLEVPQPTT 37

** :*.*.*** ***** :

Nad1_intron1 -KTNIVKEQRVDGSW-GAFNFKNIKEPLRCTLMGGESHYQIKIPSNQINKIINRNYTSNS 109

MR_cox3_intron1 -ISNIVKEQRVDGSWWIK------PIHLRCTLMGFERNYRIRIPSNQLNVKKFS------ 68

SSU_intron KISNSVKEQRVDGSYFGSLSFRGERPKLRCTLMGCENSYQIKILSKQLNNRIRT-FSHHA 96

:* *********: **** ** * *:*:* *:*:*

Nad1_intron1 YSPSPSTFPICLLDPYFVIGFSYAEVSFIVLILNLKEPKIITNWTVKIRFSIGLHKKDTE 169

MR_cox3_intron1 -----TFNYSSNVNPWFWTGLIDGEGSFTIIIDRNK--TRKLGFRVQSKFQIGVHKRDLS 121

SSU_intron KNVNVNVKMNTLLNPWFVTGFFDAEASFHISINENK--KYDLGWSVYSKFQIGLHKRDLP 154

. ::*:* *: .* ** : * . * . .: * :*.:* **:*

Nad1_intron1 ILELIKFYFGGAATISGQNEKKVFN--IE------------------------------- 196

MR_cox3_intron1 LLLQIQQFLGGIGSIYIHPTRNIVNYSVDSNKDLTNL-INHFEKYPLLTKKAADFILFKE 180

SSU_intron LLLQLQQFFCGVGQINIDSINERANFSITKISDLNNIIIPHFNEYPLQTQKTADFMLFKK 214

: :: :: * . : . .: * :

Nad1_intron1 ------------------------------------------------------------ 196

MR_cox3_intron1 VVKLMNNKAHLSIEGLHQIINIKASMNLGLSDFLKSEFNEFTPVERPVINTENIPDPNWI 240

SSU_intron AINIMLNKGHLSIEGLHRIINIKASMNLGLNDKLKSEFSNNFPVERLKFLIDNIPNPNWI 274

Nad1_intron1 ------------------------------------------------------------ 196

MR_cox3_intron1 AGFVTGEGSFDVNIPQS-TNKIGHRVQLRFRITQHERDIKLMEYLIKYLGSGKIYKYPRN 299

SSU_intron SGFVSGEGNFDVKIQKSKSHKIGYQVLLRFRISQHDKDIELMEILIKFLGTGQIEKDPRN 334

Nad1_intron1 ------------------------------------------------------------ 196

MR_cox3_intron1 AAVSLTIVKFSDITNTIIPFFEKNPLLGVKLFDYLDWCKIAKLMNDGSHLTIEGLNLIRT 359

SSU_intron SVVTLVITKFSNINNIIIPFFEKYPIQGVKQLDFLDWCKVCKLMNEGQHKKSEGLDLIRL 394

Nad1_intron1 -------------- 196

MR_cox3_intron1 IKSRMNTGRNISNI 373

SSU_intron IKTGMNTGRKFSDK 408

1. GIY-YIG HEGs

Cox1_intron7 ------------------------------------------------------YYIISN 6

MR_cox1_intron5 MGAVFGLFAGFYFWTPKILGKLYNEFLGKVHFWTLFVGVNLTFFPQHFLGMAGMYEITSN 60

Cox1_intron5 -----------------------------------------------------MT---D- 3

LSU_intron1 ----------------------------------------------MFLTNQKRK---S- 10

.

Cox1_intron7 FIENNLENSFIVSDYLLQQYYNINILV-SGTLSVTTVKYNGPHLLPKFLTSPVRIYSPN- 64

MR_cox1_intron5 LILNNLENNL------------------NLAFNLSSIIYYGPHLNPKFLKDPIRLYQPN- 101

Cox1_intron5 -----NS-----------NFFNINNYLFLFNSSAVPLTSYLSKVV--AEIKPIKVYNNFK 45

LSU_intron1 -----KG-----------KMINLNNLSI----NIIILTRNLSRGLPKLTNSPILTYNNAE 50

. : : .*: *.

Cox1_intron7 LNRNLIGVQNRKRTIIYQWINLINGKIYVGSGWNGSMRLLSYWRHSIL----TRNLPIYN 120

MR_cox1_intron5 LNRNLIGVENRKRTIIYQWFNLINSNIYVGSGWNGSFRLLSYWAPSVL----KKNLPIYN 157

Cox1_intron5 EDKQDIKKDQKDKTGVYCLINLINGNIYIGSSVNLAVRMSNYLNTTFLKNRKNNNMPIIQ 105

LSU_intron1 ELKSLIFKENLNKSFVYRWTNKVNGKTYLGSTSNAKSRLQTYYDNYTL---NLINMPIYK 107

:. * :: .:: :* * :*.: *:** * *: .* * *:** :

Cox1_intron7 SLNKYGHNNFILAILEDLGPSGSVTKSYMLNREQFYLDILFSKYPMLKLNNSPTAGSTLG 180

MR_cox1_intron5 SIVKYGHNNFCLAILEDLGPTGSVSKLYMLQREQYYLDIIFNNDSYSKLNLSPSAGTTLG 217

Cox1_intron5 ALLKYGQENFSVLIVEYVNIE------NLSVRETYYITHLLPYYNVLK-----QGYSSIG 154

LSU_intron1 AILKYGHSNFILDIIEYCDSA------ETIQKEQYYLDRFDFDYNILE-----KANSSLG 156

:: ***:.** : *:* . :* :*: : : . :::*

Cox1_intron7 FKHKEEFRLNHSGKLNPMYNKTFSTEFKNMQIRNKVGINNPQFGVKKSAETIAKLTKLIY 240

MR_cox1_intron5 FKHSEQFKLNRTGKLNPMYGREFSS----------------------------------- 242

Cox1_intron5 YKHTEATKQMLSELAKN---RTHSDKTKTLISKALVGENNPFYNKNHSMETKLRMIEANS 211

LSU_intron1 YKHNSQTIKKMKGRQNF------------------L-----GYKHTEETKDKLRELQTNK 193

:**.. . :

Cox1_intron7 ---VYEFETKN----------LIGSYSTVQCS-KEFKIGKDTLTKYIRNGIPYKNK---- 282

MR_cox1_intron5 ------------------------------------------------------------ 242

Cox1_intron5 RYSIYIYNSFKD---------LLIIFPSVNTLAKLIHSNHSSLVSYIKNKALFRGEWYLS 262

LSU_intron1 KYSVEDLEKMREIWAKRKFNSLNTNLQDLNTKTIIETESEGYVYDFVDT----------- 242

Cox1_intron7 ------------------------------------------------------------ 282

MR_cox1_intron5 ------------------------------------------------------------ 242

Cox1_intron5 NLPFNIEDTPLISNWDSKEANNLILEINNNSHIKKAIFVY-NNNYEFIKKFEGVTHAQRK 321

LSU_intron1 -------DQKL------------KLQ-KNRKKIKGKIVVVTNIKSNVSTEYISISEAASY 282

Cox1_intron7 ------------------------LFSRIKLH----------------- 290

MR_cox1_intron5 ------------------------------------------------- 242

Cox1_intron5 LNINHDIIKKYALLN---RPYKDYIFSYERLRDQFFFTFTFTNKDKVSD 367

LSU_intron1 LNITRTTLRTYMKNKTVLNILKQNPSGYGIMKEQFIINVKGK------- 324
