# Supplementary material for: Comparative structural analysis on the mitochondrial DNAs from various strains of Lentinula edodes
Source: Front Microbiol. 2022 Nov 28;13:1034387. doi: 10.3389/fmicb.2022.1034387 (PMC9744193; doi:10.3389/fmicb.2022.1034387)
Supplement: Supplementary file 3 [file Data_Sheet_3.DOCX]

**Supplementary Table 1**. Intron insertion sequences in mtDNA genes

| Intron name | Intron insertion sequences | HEGs | Group of intron insertion sequence published in Megarioti and Kouvelis (2020) |
| --- | --- | --- | --- |
| *cox1* intron1 | AGGTTTTGGT-AATTATTTTT (*L. edodes*)  TGGTTTTGGT-AACTTCTTAT (*P. citrinum*) | LAGRIDADG | IIb |
| *cox1* intron2 | AACTGTATAT-CCTCCATTAG (*L. edodes*)  GATGAACATT-CCTCCATTAT (*P. citrinum*) | LAGRIDADG | XVIII |
| *cox1* intron3 | GTTCTTTGGT-CATCCAGAGG (*L. edodes*)  ATTCTTCGGA-CACCCTGAGG (*P. citrinum*) | LAGRIDADG | VII |
| *cox1* intron4 | TACAGCTGCA-ACAATGGTAA (*L. edodes*)  TTCAGCAGCT-ACTTTAATTA (*P. citrinum*) | LAGRIDADG | Ib |
| *cox1* intron5 | TATTGGTGGA-GTAACAGGAG (*L. edodes*)  AATAGGTGGT-TTAAGTGGAG (*P. citrinum*) | GIY-YIG | III |
| *cox1* intron6 | ATTCCACGAT-ACATATTACG (*L. edodes*)  ATTCCACGAT-ACTTACTATG (*P. citrinum*) | LAGRIDADG | V |
| *cox1* intron7 | AATGGCCGGT-ATGCCTAGAA (*L. edodes*)  TTTACAAGGA-ATGCCTAGAA (*P. citrinum*) | GIY-YIG | V |
| *rrnS* intron | GAAATCCCTG-TTA/GTATATTT | LAGRIDADG | ? |
| *rrnL* intron1 | TTAATAGCGGTCT-TAACCATGAGGAT | GIY-YIG | III |
| *rrnL* intron2 | TAACCATGAGGAT-CCTAAGGTAGCA | LAGRIDADG | ? |
| *A. bisporus* *rrnL* intron2 | CCTAAGGTAGCA-GGGACGGGAAG | LAGRIDADG | XVIII |
| *cox1* intron4 in MF774813.1  (Group II intron) | A**ATTAAAAT**-----------------------**ATTCTCTTG** (*L. edodes*)  A**ATTAAAAT**ATGGGC-2,641 NTs-GACCCA**ATTCTCTTG** (*L. edodes* MF774813.1)  A**ATAAAAAT**TTGGGC-3,226 NTs-GACCCA**ATTCAGTTG** (*H. speciosa*)  G**ATCAAAAT**CTGGGC-2,581 NTs-GACCCA**ATTCTCTTG** (*A. thiersii*)  T**ATTAAAAT**ATGGGC-3,237 NTs-GACCCA**ATTTAGTTG** (*L. corticola*) | | |

**Supplementary Table 2**. Transposons in the mtDNAs of *Lentinula edodes*

|  | Retrotransposon (bp) | | DNA tranposon (bp) | Total length (bp) | mtDNA length (bp) | transposon in mtDNA  (%) |
| --- | --- | --- | --- | --- | --- | --- |
|  | LTR | NonLTR |  |  |  |  |
| MF774813 | 7465 | 756 | 4506 | 12727 | 115,116 | 11.06 |
| L808_MT | 8135 | 402 | 5133 | 13670 | 121440 | 11.26 |
| Cham_MT | 9199 | 584 | 4233 | 14016 | 121617 | 11.52 |
| Dasan_MT | 7720 | 401 | 5855 | 13976 | 121506 | 11.50 |
| Chunjang_MT | 7732 | 401 | 5489 | 13622 | 121504 | 11.21 |
| Chunbaek_MT | 8876 | 623 | 4632 | 14131 | 121641 | 11.62 |
| Baekhwa_MT | 7858 | 401 | 5095 | 13354 | 121491 | 10.99 |
| SJ707_MT | 8639 | 302 | 4616 | 13557 | 121487 | 11.16 |
| SJ701_MT | 8308 | 401 | 4668 | 13377 | 121489 | 11.01 |
| Suhyang_MT | 8227 | 401 | 4776 | 13404 | 121489 | 11.03 |
| AB697988 | 7911 | 400 | 4707 | 13018 | 121394 | 10.72 |
| SL7_MT | 8145 | 295 | 4314 | 12754 | 121487 | 10.50 |
| SJ302_MT | 8210 | 302 | 4632 | 13144 | 121486 | 10.82 |
| SJ301_MT | 8349 | 302 | 4823 | 13474 | 121486 | 11.09 |
| Gaeul_MT | 7964 | 407 | 5059 | 13430 | 121440 | 11.06 |
| Yeoreum_MT | 8115 | 489 | 4417 | 13021 | 121491 | 10.72 |
| MF774812 | 8133 | 642 | 4522 | 13297 | 119,134 | 11.16 |
| Yujiro_MT | 7547 | 537 | 4474 | 12558 | 119233 | 10.53 |
| SJ102_MT | 7698 | 247 | 4260 | 12205 | 121671 | 10.03 |
| SMR1_MT | 7315 | 626 | 5278 | 13219 | 121299 | 10.90 |
| Pungnyun_MT | 7865 | 411 | 5040 | 13316 | 121508 | 10.96 |
| SL10_MT | 7700 | 576 | 5297 | 13573 | 119219 | 11.38 |
| SL9 | 7927 | 517 | 4381 | 12825 | 121481 | 10.56 |
| L54_MT | 9027 | 640 | 4470 | 14137 | 119500 | 11.83 |
| KY217797 | 8017 | 446 | 4782 | 13245 | 116897 | 11.33 |
| Average | 8083.28 | 460.36 | 4778.36 | 13322 | 120700.2 | 11.04 |

**Supplementary Table 3**. Major transposon contents in *Lentinula edodes* mtDNAs

|  | hAT | | Copia | | Gypsy | |
| --- | --- | --- | --- | --- | --- | --- |
|  | length (bp) | count | length (bp) | count | length (bp) | count |
| AB697988 | 3,694 | 23 | 1,753 | 14 | 6,158 | 53 |
| SL7_MT | 3,462 | 19 | 1,701 | 13 | 6,320 | 51 |
| SL9_MT | 2,839 | 20 | 1,651 | 13 | 6,276 | 54 |
| SL10_MT | 4,245 | 23 | 1,785 | 14 | 5,915 | 53 |
| Yeoreum_MT | 3,331 | 22 | 1,754 | 14 | 6,237 | 52 |
| Gaeul_MT | 3,972 | 24 | 1,754 | 14 | 6,086 | 54 |
| Baekhwa_MT | 4,078 | 26 | 1,903 | 15 | 5,955 | 51 |
| Dasan_MT | 4,494 | 26 | 1,903 | 15 | 5,817 | 52 |
| Cham_MT | 3,147 | 20 | 1,754 | 14 | 7,445 | 61 |
| KY217797 | 3,446 | 22 | 1,854 | 15 | 5,980 | 50 |
| Suhyang_MT | 3,712 | 22 | 1,754 | 14 | 6,349 | 52 |
| Chunbaek_MT | 3,545 | 22 | 1,754 | 14 | 7,122 | 59 |
| Pungnyun_MT | 3,839 | 24 | 1,785 | 14 | 6,080 | 53 |
| Chunjang_MT | 4,128 | 25 | 1,754 | 14 | 5,978 | 53 |
| SMR1_MT | 4,078 | 27 | 1,783 | 14 | 5,408 | 47 |
| L54_MT | 3,422 | 20 | 1,795 | 14 | 7,232 | 58 |
| Yujiro_MT | 3,523 | 21 | 1,686 | 13 | 5,861 | 49 |
| L808_MT | 4,230 | 25 | 1,865 | 15 | 6,270 | 56 |
| SJ102_MT | 3,291 | 22 | 1,644 | 13 | 6,054 | 50 |
| SJ701_MT | 3,654 | 22 | 1,754 | 14 | 6,430 | 53 |
| SJ707_MT | 3,712 | 22 | 1,852 | 15 | 6,663 | 55 |
| SJ301_MT | 3,919 | 22 | 1,975 | 16 | 6,250 | 52 |
| SJ302_MT | 3,728 | 21 | 1,975 | 16 | 6,111 | 51 |
| MF774812 | 3,508 | 20 | 1,686 | 13 | 6,447 | 52 |
| MF774813 | 3,283 | 19 | 1,555 | 15 | 5,597 | 51 |
| Average | 3,691 | 22 | 1,777 | 14 | 6,242 | 53 |
