# Supplementary figures and images for: Comparative structural analysis on the mitochondrial DNAs from various strains of Lentinula edodes
Source: Front Microbiol. 2022 Nov 28;13:1034387. doi: 10.3389/fmicb.2022.1034387 (PMC9744193; doi:10.3389/fmicb.2022.1034387)

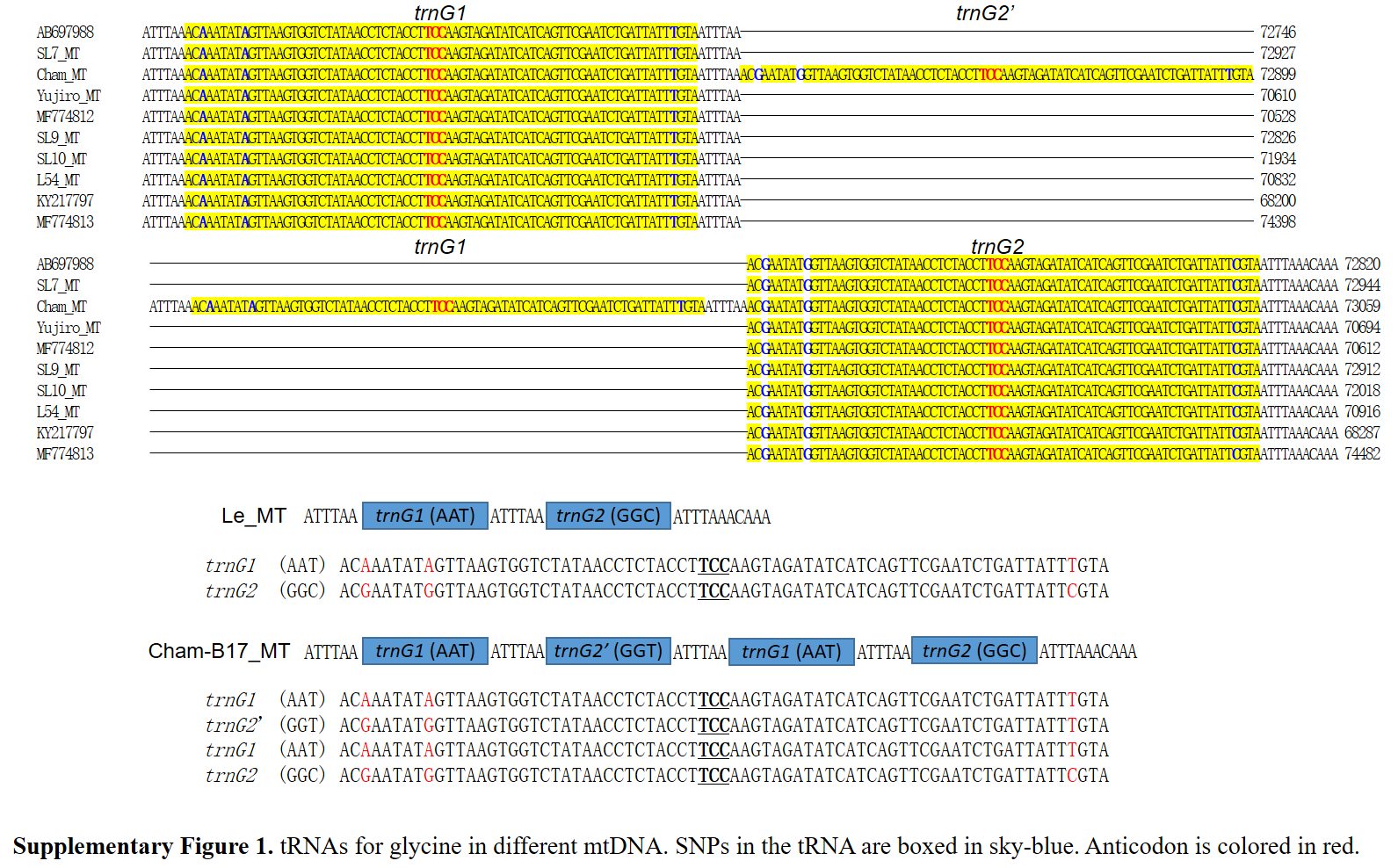

Supplement: Supplementary file 4 [file Image_1.TIF]

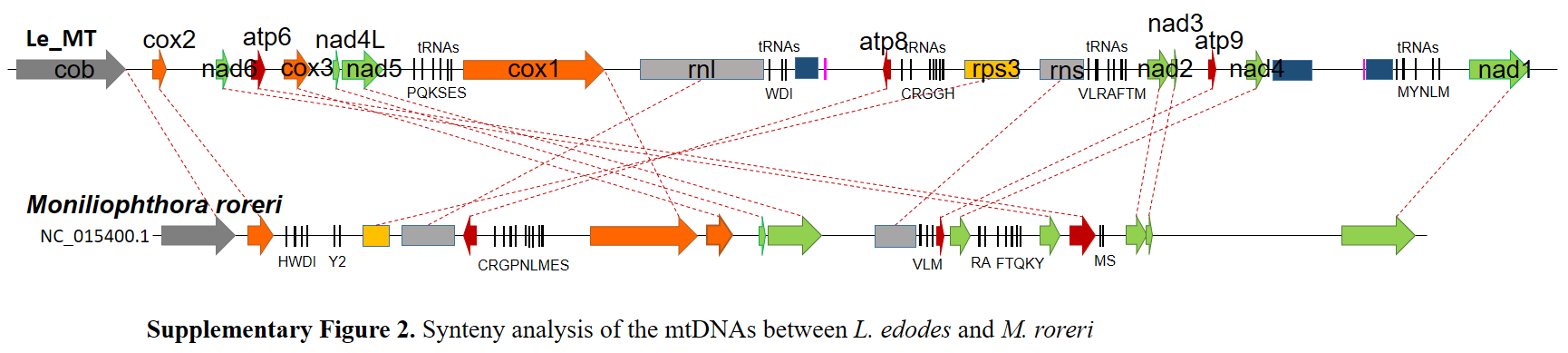

Supplement: Supplementary file 5 [file Image_2.TIF]

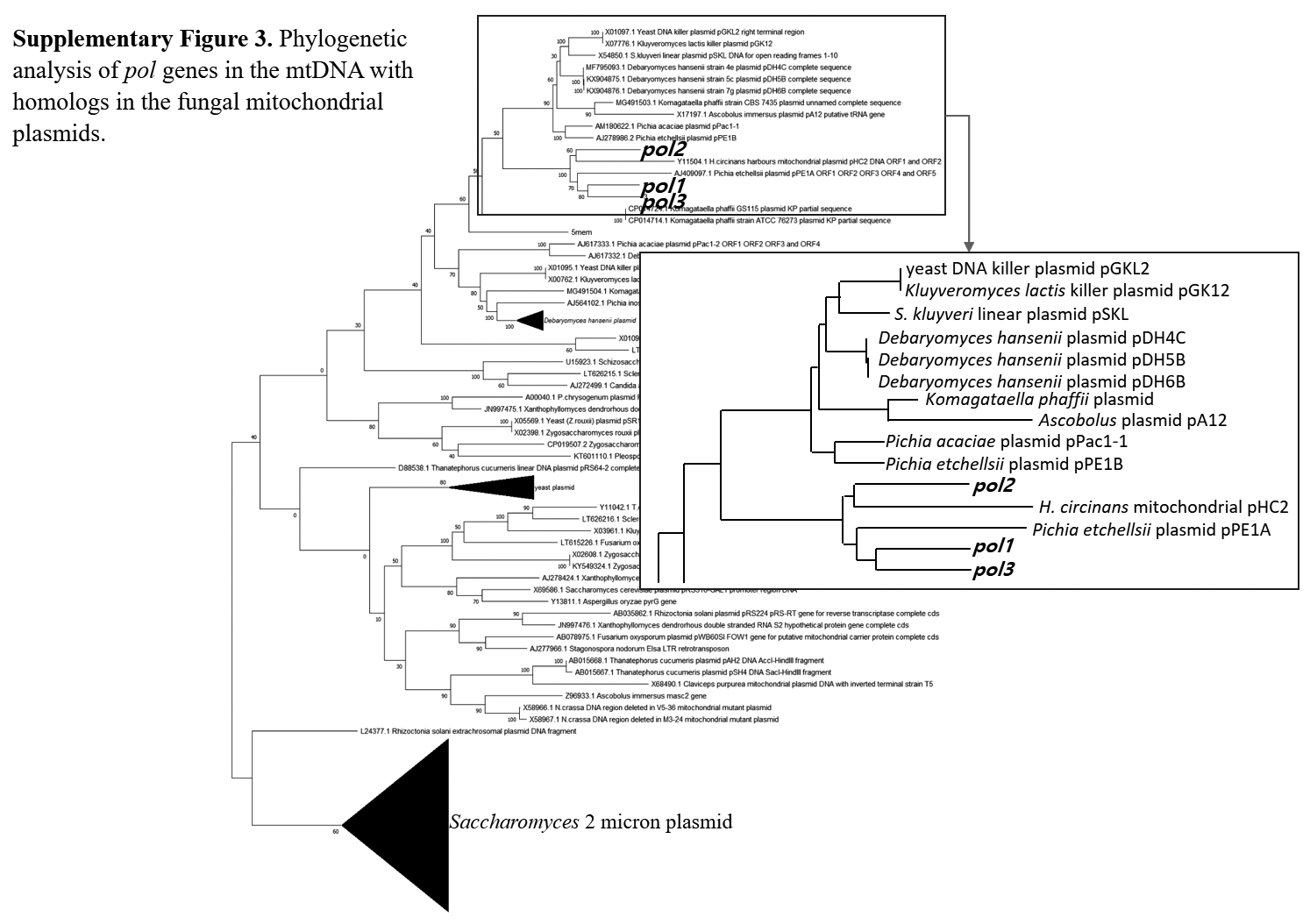

Supplement: Supplementary file 6 [file Image_3.TIF]
